# ReSpecTh: Reaction kinetics, spectroscopy, and thermochemical datasets

**DOI:** 10.1038/s41597-025-05272-6

**Published:** 2025-06-17

**Authors:** Tamás Turányi, István Gy. Zsély, Máté Papp, Tibor Nagy, Tibor Furtenbacher, Roland Tóbiás, Péter Árendás, Attila G. Császár

**Affiliations:** 1 Institute of Chemistry, ELTE Eötvös Loránd University, H-1117 Budapest, Pázmány Péter sétány 1/A, Hungary; 2 HUN-REN Research Centre for Natural Sciences, Magyar Tudósok körútja 2, 1117 Budapest, Hungary; 3 Budapest University of Economics and Business, Budapest, Hungary

**Keywords:** Reaction kinetics and dynamics, Infrared spectroscopy

## Abstract

Description of a large number of datasets related to gas-phase reaction kinetics (Re), high-resolution molecular spectroscopy (Spec), and thermochemistry (Th), called ReSpecTh, is presented. The datasets contain accurate and validated experimental, empirical, and computed, machine-searchable data, and, whenever possible, the corresponding metadata. ReSpecTh data and the accompanying utility codes can be used in several engineering and scientific fields either separately or simultaneously, such as simulation of combustion reactions, atmospheres of planets and exoplanets, and stellar and interstellar environments.

## Background & Summary

Knowledge of accurate data, and related metadata, has an eminent place in modern science and engineering, a statement true for many fields of chemistry. In the fourth age of quantum chemistry^1^, the availability of high-level, complex algorithms and related sophisticated computer codes makes it feasible to (a) validate experimental data, (b) generate new, empirical data augmenting existing measured data, and (c) use experimental, empirical, and computed data on a common basis to help diverse scientific and engineering applications. This paper provides a description of the result of our efforts to collect published experimental kinetic, high-resolution spectroscopic, and thermochemical data and to include them in datasets under the common name ReSpecTh. The aim of the ReSpecTh project has been to help to (a) understand our wider natural environment (*e.g*., star-forming regions, the interstellar medium, the atmospheres of planets and exoplanets), (b) answer important questions related to the origin of life on Earth and the special role of humans in the preservation of life’s diverse forms, (c) improve our understanding of complex chemical reaction systems, (d) protect the quality of life on our planet, (e) validate and develop gas-phase kinetic reaction mechanisms to understand and model processes in combustion and atmospheric chemistry, (f) develop more efficient, low-emission combustion devices for both current and novel fuels, and (g) assist air-quality prediction and emission limit settings (*e.g*., *via* an improved scientific understanding of the greenhouse effect and of combustion systems, as well as research and development related to climate change).

An improved understanding of the various processes and environments mentioned requires the availability of carefully validated data of traceable origin. Indeed, several databases and information systems exist in the fields of reaction kinetics^2–10^, high-resolution molecular spectroscopy^11–17^, and thermochemistry^18–29^. About a decade ago, we set up a dedicated website, https://respecth.hu/, freely accessible *via* a login system after registration, containing a large number of datasets in reaction kinetics, high-resolution molecular spectroscopy, and thermochemistry. At present, the number of registered users of ReSpecTh is close to 1000. After a decade of development, this paper describes the ReSpecTh datasets, publicly available at an OSF (Open Science Framework) repository, https://osf.io/nbmzv/^30^.

The ReSpecTh datasets have several unique and noteworthy features. The kinetic branch contains searchable datasets of gas-phase chemical kinetics, focusing on elementary reactions relevant to combustion. It includes detailed experimental data and tools for assessing the uncertainty of reaction rate parameters. The spectroscopic datasets are built upon data mining, using methods of discrete mathematics novel to molecular physics, such as graph (network) theory. Many of the collected experimental and computed data influence the values and the uncertainties of the derived (empirical) data. It is also important to note that a considerable part of the thermochemical data are based on detailed spectroscopic line-by-line information.

Creation of the ReSpecTh datasets followed the FAIR (Findable, Accessible, Interoperable, and Reuseable) principle^31,32^. The measured and theoretically determined data in our datasets are *findable* thanks to sophisticated, detailed search engines designed for user convenience. Each XML file has a unique identifier referenced throughout the database. In the case of chemical kinetics data, for example, the core of a measurement data file is formed by measured ignition delay times (IDT), flame velocities, laminar burning velocities (LBV), and temperatures or concentrations. Each data file is rich with metadata describing in detail the measuring device, includes a link to the original publication containing the measurement data (BibTeX fields and DOI link) along with the CAS, InChI, and SMILES identifiers of the chemical substances measured. Within the spectroscopy datasets each source has a standardized unique identifier, composed from the year of the publication and the first letters of the names of the authors. Each transition of each source also receives a unique identifier. The data are *accessible* at the OSF repository^30^; furthermore, the ReSpecTh website provides easy downloads by data file, both individually and in groups after a free registration using the user’s login credential. *Interoperability* of data is also ensured, because the files store the data in a well-described, standardized format. In the case of the kinetic data this is the ReSpecTh Kinetics Data (RKD) Format^33^, while in the case of the spectroscopic data the format required by the MARVEL (Measured Active Rotational-Vibrational Energy Levels) code^34,35^. The XML-based RKD data format is standalone, and can be used outside our environment. There are already existing tools^36,37^ that can convert these files into input files of popular chemical kinetic modelling program packages^37–40^. Optima++^36^ can also convert the RKD-format data files to plain text files. The data can be *reused* because the meaning of each tag used within ReSpecTh is described, allowing the users to understand, interpret, or even re-code the information. Correcting errors or adding new information in the formats applied is well handled and these changes do not invalidate the unmodified part of the files.

The rate of data accumulation in the fields of combustion research, as well as of reaction kinetics, has been steadily increasing: the current literature contains an extremely large number of experimental combustion data and detailed combustion mechanisms^41^. This leads to typical problems of ‘big data’: storage, search, and sharing. Sometimes it is argued that a combustion database based on direct and indirect experimental data is not of ‘big data’ type, since the amount of data for a given fuel and at a given set of conditions is limited. However, because of the hierarchical nature of the combustion chemistry of fuels^42^, the experimental data for hydrogen, syngas, methane, ethane *etc*. are also applicable for the validation of the high-temperature combustion mechanism of a higher hydrocarbon, like propane. Furthermore, there is a demand for mechanisms applicable in wide ranges of conditions (temperature, pressure, and equivalence ratio), which requires a comprehensive set of data.

Traditional scientific papers have been ideal for sharing new ideas and describing procedures and methods, but they are far from being ideal for storing data. In these papers, neither the figures and the tables, nor the typical supplementary files are in machine-readable form. The common search engines, designed to explore the scientific literature, can find information by keywords, but not by the experimental data themselves. These difficulties lead to poor and ineffective access to measurement data in general, and in reaction kinetics in particular.

Only a few databases contain information on the determination of direct experimental and theoretical reaction rate coefficients. The most famous and comprehensive one is the NIST Chemical Kinetics Database^2^. This contains more than 38 000 separate reaction records for over 11 700 distinct reactant pairs, abstracted from over 12 000 papers. The website of an IUPAC Task Group on ‘Atmospheric Chemical Kinetic Data Evaluation’^3^ provides evaluated kinetics and photochemical data, related to a wide range of atmospheric chemical processes. The EUROCHAMP-2020 database^4^ has a much narrower focus: it provides a compilation of recommended rate coefficients for reactions of volatile organic compounds with the OH and NO_3_ radicals, ozone, and Cl atoms under atmospheric conditions. The JPL database^43^ provides a series of evaluated sets of rate constants, photochemical cross sections, heterogeneous parameters, and thermochemical parameters compiled by the NASA Panel for Data Evaluation. The Master Chemical Mechanism^44^ contains information on detailed gas-phase chemical processes involved in the tropospheric degradation of a series of primary emitted volatile organic compounds (VOCs). Currently, the degradation of methane and 142 non-methane VOCs is represented.

In the field of combustion kinetics, Frenklach^45^ suggested collaborative data processing for the analysis and development of detailed reaction mechanisms. Frenklach emphasized^46^ that the simultaneous application of large amounts of experimental data for testing a combustion mechanism may provide a new level of understanding. The related informatics infrastructure was called PrIMe (Process Informatics Model). Unfortunately, neither the original PrIMe website^6^, nor its local version at DLR Stuttgart are functioning. The PrIMe collection contained interlinked experimental, bibliographic, and species information data. The PrIMe experimental data format defined limited types of experimental data and new formats were not defined to accommodate other data types.

KAUST Cloudflame^7^ is a cloud-based cyberinfrastructure for managing combustion research and enabling collaboration. The infrastructure includes both software and hardware components and is freely offered to anyone with a valid professional or educational affiliation. This website provides a front-end for data-search tools, web-based numerical simulations, and discussion forums. It includes a collection of combustion data, which mainly focuses on liquid fuels and is limited to jet-stirred reactors (JSR) and ignition-delay-time (ST-IDT) measurements. There is no search engine for experimental conditions, and the data files use the csv format and an earlier version of the ReSpecTh Kinetics Data Format (RKD, see below). The last data uploads occurred in 2019.

The SciExpeM database^47,48^ contains more than 38 000 indirect experimental data points in more than 2000 RKD format data files. The main aim of this database is to develop simulation models for the combustion kinetics of green fuels related to the Industry 4.0 concept.

ChemKED^8,49^ is a GitHub repository limited to IDT measurements. The data files are handled by PyKED, a Python-based software applicable for validating and interacting with ChemKED files. There is no search engine connected to these data. A major update took place in 2018, with limited updates in 2021.

The UConn Combustion Database^9^ contains the data measured in the Combustion Diagnostics Laboratory of the University of Connecticut (UConn). This data collection contains IDTs measured in rapid compression machines (RCM) and laminar burning velocity measurements. The data are in Excel tables and the related data are provided in downloadable zip files. There is no search engine for these data.

The Fundamental Kinetics Database Utilizing Shock Tube Measurements^10^ summarizes the published shock tube experimental measurements performed under the supervision of Prof. Hanson. The database covers the years from 1974 to 2019 inclusively. The database is a collection of ignition delay time, speciation, and rate measurements in shock tubes, with data given in MS Word and PDF files. There is no search engine for these data, and computer reading of such files cannot be straightforwardly automated.

There are few comprehensive databases of detailed combustion mechanisms available on the internet. GITHUB hosts minor collections of combustion mechanisms in CHEMKIN format^50–52^. The SciExpeM web site^47^ contains 207 mechanisms for 30 fuels. There are websites containing mechanisms developed within a research group. These websites are maintained by the Combustion Chemistry Centre, University of Galway, Galway, Ireland^53^, the CRECK Modeling Group, POLIMI, Milano, Italy^54^, the Combustion Research Group, UCSD, San Diego, CA^55^, the Combustion Kinetics Laboratory, USC, Los Angeles, CA^56^, the Clean Combustion Research Center, KAUST, Kingdom of Saudi Arabia^57^, and LLNL, Livermore, CA, USA^58^.

There are a few websites which contain utility codes for the analysis and development of detailed combustion mechanisms. The LLNL Mech Checker website^59^ provides tools^60^ to improve the performance of chemical mechanisms. The site contains the Thermodynamic Checker and the Ignition Delay Time Diagnostic tool. The Thermodynamic Checker (a) refits the NASA polynomial coefficients to correct for the discontinuities in the thermodynamic functions between the lower and upper temperature ranges of their definition, and (b) plots thermodynamic data for isomers, so the user can determine if there are any outliers. A similar tool is provided by the authors of OpenSMOKE++^37^. The Ignition Delay Time Diagnostic tool utilizes the Zero-RK solver to run single ignition delay calculations. The results are then analyzed to provide the user with information about the mechanism and flag expected problem areas. A similar web tool is available at the CloudFlame website^7^ for screening gas-phase chemical kinetic models based on collision limit compliance.

Clearly, the availability of a searchable database of experimental combustion data, direct rate coefficient determinations, and combustion mechanisms should significantly assist combustion research. One of the aims of creating the reaction kinetics branch of ReSpecTh was to provide a hub, an all-in-one place, for measurements, rate coefficient determinations, reaction mechanisms, and tools for combustion simulations.

Note that databases of different types are also of high value in combustion science. For example, there is an effort to create databases of DNS simulation results of turbulent flames (see, *e.g*., the BLASTNET^61^ network).

In the field of high-resolution rovibronic spectroscopy, several data repositories have been set up (for example, in alphabetical order, CDMS^14^, GEISA^13^, HITRAN^16^, and JPL^17^, as well as several others concerning individual molecules and their isotopologues, such as the carbon dioxide spectroscopic database CDSD-296^15^). These databases contain measured or empirically calculated, mostly rovibrational transitions for a number of molecules, typically in various formats and with even more different verification procedures. Thus, there is a need for standardized database files, which can be validated by well-established protocols, like MARVEL (Measured Active Rotational-Vibrational Energy Levels)^34,35^.

Spectral lines are described by a number of parameters: position, intensity, width, shape, labels referring to the upper and lower quantum states^62^, as well as self and foreign shift/ broadening due to collisions with many different partners. Of all this information, at present we aim to store and validate only line positions, uncertainties, and assignments.

The spectral line parameters may undergo significant changes over time as more modern measurement techniques are made available, improving sensitivity, resolution, precision, and overall accuracy. Measurements of gradually increasing accuracy not only substantially alter the positions and the uncertainties of the individual lines, but often result in assignment changes, as well. Numerous conflicts have been detected among data reported by different experimental groups due to the use of inconsistent labels. Unfortunately, the assignment of spectral lines using first-principles computations remains a significant challenge even today^63^, especially at higher excitation. Hence, there is a considerable need for developing algorithms capable of verifying and merging a large number of measured and assigned lines. In 2007, an IUPAC Task Group decided to attempt to refine our understanding about the measured rovibrational spectra of one of the (if not the) most important molecules, water. One of the outcomes of this effort was the MARVEL algorithm^34,35,64–66^. Through an inversion process, MARVEL inverts the information contained in the measured lines, yielding empirical rovibronic energy levels. Then, the task is to ensure that all the experimental, empirical, and computed spectroscopic information available is self-consistent and the data put into a spectroscopic database are as complete as possible.

The MARVEL approach has been used to analyze a large number of spectra measured for a large number of molecules, including diatomics (^12^C_2_^67,68^, ^12^CH^69^, ^14^NH^70^, ^16^OH^69^, ^16^O_2_^71^, ^48^Ti^16^O^72^, and ^90^Zr^16^O^73^), triatomics (several isotopologues of carbon dioxide^74,75^, ${{\rm{H}}}_{3}^{+}$^76^, H_2_D^+^ and D_2_H^+^^77^, nine water isotopologues^78–86^, two isotopologues of H^16^OCl^87,88^, ${{\rm{H}}}_{2}^{32}$S^89^, ^14^N_2_^16^O^90^, and four sulphur isotopologues of S^16^O_2_^91^), tetratomics (^12^C_2_H_2_^92^, ${{\rm{H}}}_{2}^{12}{{\rm{C}}}^{16}$O^93^, and ^14^NH_3_^94,95^), and even a pentatomic molecule, ${{\rm{H}}}_{2}^{12}{{\rm{C}}}_{2}^{16}{\rm{O}}$^96^.

For stable molecular species, a number of thermochemical data compilations are available^20–24,27,28^, utilized in a number of scientific and engineering applications. These databases are characterized by rather different levels of accuracy and occasionally contain results which do not agree within the stated uncertainties. The most important thermochemical data are the enthalpies of formation, the bond dissociation energies, the reaction free energies, and the temperature-dependent ideal gas partition functions, *Q*(*T*), and quantities which can be derived straightforwardly from *Q*(*T*)^97–100^.

Enthalpies of formation and heat capacities of stable molecules have traditionally been determined by calorimetry. Derivation of some additional thermochemical quantities, such as entropies, has been based on results of detailed spectroscopic measurements. Short-lived intermediates are usually not amenable to direct calorimetric measurements, and their spectroscopic characterization also requires special efforts and sophisticated instrumentation. Thus, experimental thermochemical data for a large number of species of interest is either missing from the databases or have relatively large uncertainties (or their uncertainties are significantly underestimated)^101^.

A new era in the treatment and validation of thermochemical data came with the introduction of the ATcT (Active Thermochemical Tables) approach^25,28,29,101^, yielding high-quality empirical thermochemical quantities, in particular enthalpies of formation, as a function of temperature. The ATcT approach has been used successfully in a number of cases, including the determination of the enthalpies of formation of radicals^26^.

The recent blossom of research in the field of thermochemistry is due to several important developments. Besides new measurement techniques, the ability of first-principles quantum chemistry to produce accurate thermochemical quantities has improved tremendously, even for open-shell systems and molecules containing heavier elements. Modern first-principles thermochemistry relies on advanced techniques of electronic-structure theory, allowing the computation of accurate thermochemical quantities with well-defined uncertainties^26,102^. For the determination of temperature-dependent, effective quantities, one should use variational-like techniques of nuclear-motion theory, readily available in the fourth age of quantum chemistry^1^. One of the key questions in electronic-structure theory concerns the accuracy of the computed molecular quantities, including relative energies. The composite focal-point analysis (FPA) approach developed more than three decades ago by one of the authors^103,104^ provides a clean and straightforward way to determine the uncertainties of computed relative energies. It should also be noted that the accuracy of the computed first-principles enthalpies of formation can be improved significantly by the use of properly chosen reaction schemes^105^, which also offer improved capability for computational thermochemistry of larger systems.

Traditionally, the computational approaches to thermochemistry relied either on simple assumptions, like the harmonic-oscillator^106^ and rigid-rotor^107^ models, or at best on effective Hamiltonians (EH)^108^ fitted to experimental data. These modeling efforts have resulted in relatively poor predictions with unknown uncertainties, especially at higher temperatures. First-principles thermochemistry is able to determine highly accurate partition functions and related thermochemical data, with well-defined uncertainties: the direct summation technique, based on accurate rovibrational energy levels with known small uncertainties, offers a straightforward way to determine accurate thermochemical quantities over a wide temperature range (even up to temperatures of 6000 K).

According to old conventions of thermochemistry, the definition of the enthalpy of formation does not reference atomic information and the enthalpies of formation of all elements in their standard states are zero, independent of the temperature. Advantages of atom-based thermochemistry (AT) over the conventional element-based one have been pointed out several times^109–114^. One problem with the element-based convention is that the elements in their standard states may be gases, liquids, or solids of different constitution. Another one is that for ab initio quantum chemical energy and enthalpy calculations, which are bound to have an ever increasing role in the determination of thermochemical quantities, the natural reference is the atom in its ground electronic state.

## Methods

### Reaction kinetics

Experimental chemical kinetics data are called *direct* if the aim of the experiment is to determine the rate coefficient of an elementary reaction at a set of given conditions (temperature, pressure, and bath gas). A typical experimental method to obtain direct kinetic data is laser photolysis – laser-induced fluorescence detection (LP-LIF). High-level first-principles computations can also be used to determine the rate coefficient of an elementary reaction at a given temperature, pressure, and bath gas, and these results, when accompanied by proper uncertainty estimates, can be treated similarly to those of direct measurements.

Experimental kinetics data are called *indirect* if rate coefficients cannot be determined directly from them, but the measured value can be reproduced *via* simulation using a detailed reaction mechanism. The indirect combustion data in ReSpecTh contain ignition delay times, measured in shock tubes and rapid compression machines (RCMs), as well as laminar flame velocities and concentration profiles, observed using facilities like laminar and turbulent flow reactors, micro-flow reactors, jet-stirred reactors (JSRs), shock tubes, and burner-stabilized flames.

**Fig. 1:**
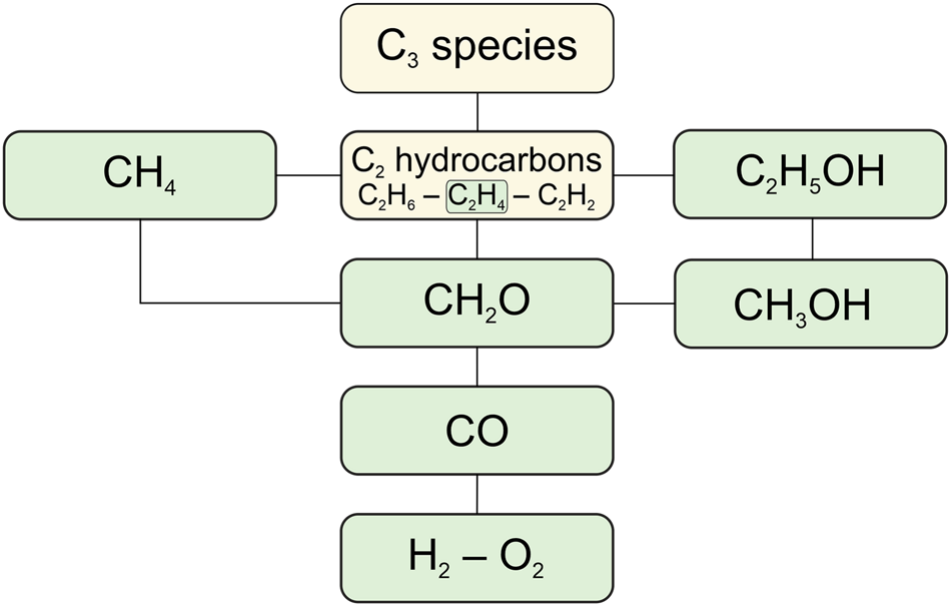
The hierarchy of hydrocarbon fuels according to Westbrook and Dryer^115^. Green shadings indicate fuels present in ReSpecTh.

Our kinetics dataset^30^ contains more than 162 000 kinetics data points in more than 3600 XML data files of indirect experimental data. The order of adding the data roughly followed the hierarchy of fuels, as defined by Westbrook and Dryer^115^ (see also Fig. 1).

#### The ReSpecTh Kinetics Data Format

In our previous studies^116–133^, the important elementary reaction steps under various conditions were identified using local sensitivity analysis and the results of several direct determinations of the corresponding rate coefficients were encoded in the ReSpecTh Kinetics Data (RKD) format. Both the direct and the indirect data files in the reaction kinetics branch of ReSpecTh were encoded in the RKD format, the latest version of which is ver. 2.5^33^.

Most of the RKD specifications are directly derived from the XML elements and attributes defined in PrIMe^6^, extended in various directions, making the two incompatible. The detailed RKD Format Specification^33^ describes not only the possible tags and attributes, but also how they should be interpreted. The RKD format (a) contains specifications which provide unambiguous definitions for the storage of experimental combustion data and rate coefficient determinations, and (b) describes an XML document type definition which allows flexible data representation and easy extension of the format specification.

Our aim with the provision of a data-format specification for combustion experiments, along with experimental and theoretical rate-coefficient determinations, was the support of long-term, laboratory-independent storage of related data and metadata. Storage of data in the RKD format allows an unambiguous interpretation of the data for simulations, as well as sharing and archiving the data in databases. Both might have very important long-term impacts on the combustion community. The unambiguous data format prevents misinterpretation and caters all the information needed to set up the related simulations. Sharing RKD format files in databases makes combustion research considerably more efficient.

According to the traditional workflow, researchers had to collect the experimental data from individual publications. Keyword-based search engines for scientific publications are not effective when someone is looking for a specific experimental condition. Even if relevant publications were found, the data had to be extracted from figures or files readable only by humans. This requires a lot of effort and is certainly error-prone. Unfortunately, even in some recent publications, the measured data are reported only in figures, or key information is missing or remains ambiguous. These potential problems are eliminated by having the experimental results reported in RKD format files.

Archiving data is also a very important issue. It occurs frequently that important details are missing from the published paper and that the research group does no longer exist. In other cases, the authors can be contacted, but the paper logs have been lost, while the electronic lab logs have been deleted, or use a format or storage media that cannot be read anymore. Therefore, it is very important to make all experimental data available in public databases, which allows the community to use them in the long run.

The XML data format allows (a) flexible data representation, (b) easy extension of the format specification, and (c) the introduction of new tags which do not invalidate the older ones. The RKD format was substantially revised once, indicated by a change from ver. 1.6 to 2.0, then it was extended several times during the last few years, indicated by changes in the first decimal of the version number. All these minor extensions enabled new features, preserving, at the same time, backward compatibility. Further extensions are planned by the addition of new types of experiments, like encoding extinction limits, soot measurements, and experimental data measured in stretched flames.

The PrIMe data format included references to the bibliographic database of the PrIMe server and the names of the species were linked to the species identification dataset of PrIMe. Therefore, using a PrIMe experimental data file required a functioning PrIMe server, allowing these connections. The RKD format was designed to work standalone. Using an RKD format file does not require any link to the ReSpecTh website. All bibliographic data are stored in XML format BibTeX fields and all publications are identified with DOI links (if they exist). This means that all original publications can be immediately inspected by clicking on the provided DOI link. The species defined in the experimental data files are identified using CAS, InChI, and SMILES descriptors, which allows straightforward linking to internet databases describing properties of these substances.

Data files obeying the RKD format contain two main sections. The first one identifies the source of the data and provides information about the creation of the file. The data source is not only referenced to the literature using BibTeX keywords, but a detailed description shows the exact location of the information within the article. There is information about the creator of the file, the version of the RKD format, the date of the first creation, and the date of the last modification. The second section is divided into a few subsections. In the indirect experimental RKD files there are three main subsections. The first one identifies the experimental setup (type of experiment, apparatus, and measurement-related keywords, *e.g*., the definition of the ignition delay time used in the measurement). The second one provides data about the experimental conditions that were kept constant during the experiment (*e.g*., pressure, temperature, equivalence ratio, and initial concentration). The third subsection provides the varied experimental condition(s) and the measured values (*e.g*., ignition delay time, laminar burning velocity, and species concentration). The proper definition of the ignition delay time should be emphasized, since the application of an inappropriate definition may lead to significant errors in simulated ignition delay times^134^. The uncertainty of the measured data can also be provided. The rate determination RKD format files (referring to direct measurements and theoretical calculations) contain two main subsections. The first one describes the elementary chemical reaction for which the rate coefficients were determined. The second subsection contains the values of the measured or calculated rate coefficients.

A valid indirect experimental RKD format file must contain all information needed to set up a simulation using the experimental data encoded. In the case of concentration measurements, one dataset may contain more than one concentration profile, when several concentrations are measured in the same experiment. These concentration profiles, called data series, are stored together in a single RKD format file. Some experiments need additional profiles to consider, *e.g*., temperature-distance profile in burner-stabilized flame experiments, or pressure-time profiles in ignition-delay-time experiments. These profiles can be stored in the same XML file.

The utility code Optima++^36^ can be used to create new RKD format data files and check the consistency of external RKD format files. Optima++ can be applied for an automatic testing of reaction mechanisms using selected datasets.

#### Detailed combustion mechanisms

The indirect combustion experimental data have been used for testing published reaction mechanisms. For the combustion of each investigated fuel, detailed reaction mechanisms, in Chemkin format^135^, were collected from the authors’ websites, the supplementary files of publications, or obtained directly from the authors. These mechanisms were also converted to OpenSMOKE++^37^ and Cantera^38^ mechanism formats, providing the transport files and the thermodynamic files in NASA polynomial format. The aim has been to acquire, for each fuel, all mechanisms published during the last 20 years. These mechanisms are related to the combustion of hydrogen^116,117^, syngas^118,119^, methanol^121^, ethanol^120^, methane (ignition delay time)^126^, ethylene^130,136^, H_2_/O_2_/NO_*x*_ mixtures^122^, syngas/NO_x_ mixtures^122^, ammonia^123,124,133^, methanol/NO_*x*_ mixtures^125^, butanol^129^, and pentane^131^ (see Table 1). Furthermore, using the collection of indirect and direct data, new, significantly more accurate reaction kinetic models were created mainly via kinetic parameter optimization for the combustion of hydrogen^117^, syngas^119^, methanol^121^, ethanol^120^, H_2_/O_2_/NO_*x*_^122^ and methanol/NO_*x*_^125^ fuels. These six optimized mechanisms can be downloaded in Chemkin^135^, OpenSMOKE++^37^, and Cantera^38^ formats. Until now, no thematic collection of combustion mechanisms existed, and the present project provides a unique collection of combustion mechanisms.

**Table 1:** Indirect reaction kinetics datasets collected and made available within ReSpecTh.

Fuel	#files	#datasets	#data points	#models	References	
					mechanism comparison	optimization
hydrogen	177	208	2327	21	Olm *et al*.^116^	Varga *et al*.^117^
syngas	387	423	4852	17	Olm *et al*.^118^	Varga *et al*.^119^
methanol	287	584	14849	19	Olm *et al*.^121^	Olm *et al*.^121^
ethanol	192	504	16808	17	Olm *et al*.^120^	Olm *et al*.^120^
methane	1294	1294	11153	14	Zhang *et al*.^126,127^	
ethylene	219	397	4323	14	Su *et al*.^130^	Su *et al*.^136^
hydrogen/NO_x_	207	332	4949	18	Kovács *et al*.^122^	Kovács *et al*.^122^
syngas/NO_x_	61	145	1834		Kovács *et al*.^125^	Kovács *et al*.^132^
methanol/NO_x_	74	243	2574	21	Kovács *et al*.^125^	Kovács *et al*.^132^
ammonia	351	527	5278	44	Kawka *et al*.^123^	
					Szanthoffer *et al*.^124,133^	
butanol	266	475	89347	29	Bolla *et al*.^129^	
pentane	72	226	3629			Wang *et al*.^131^

#### Utility codes

A series of computer codes are available in ReSpecTh which can be used for the analysis, reduction, and optimization of detailed reaction mechanisms, for sensitivity and uncertainty analyses, as well as for the quantification of the uncertainty of experimental data and model parameters. In most cases, the codes are available as source codes, accompanied by manuals and sample input/output files. We also host several similar computer codes that were developed by authors we have collaborated with. Most codes have both Windows and Linux versions.

Optima++ is the central code in the reaction kinetics branch of ReSpecTh. It has a command-line version and a GUI-based version. Optima++ reads the data provided in text files and prints out XML data files in proper RKD format. The code checks the data for completeness (whether all physical conditions are provided and the experiment is well-defined) and identifies possible contradictions (*e.g*., whether the reaction temperature was defined multiple times). Optima++ itself cannot be used to perform combustion simulations, but it reads RKD format data files, and sets up input files for simulation codes FlameMaster^39^, OpenSMOKE++^37,137^, Cantera^138^, or Zero-RK^40^. Optima++ is able to run these simulation packages on the same PC, on an external PC, or on an external computer cluster. Then, Optima++ reads the output files of the simulation codes and processes them.

**Fig. 2:**
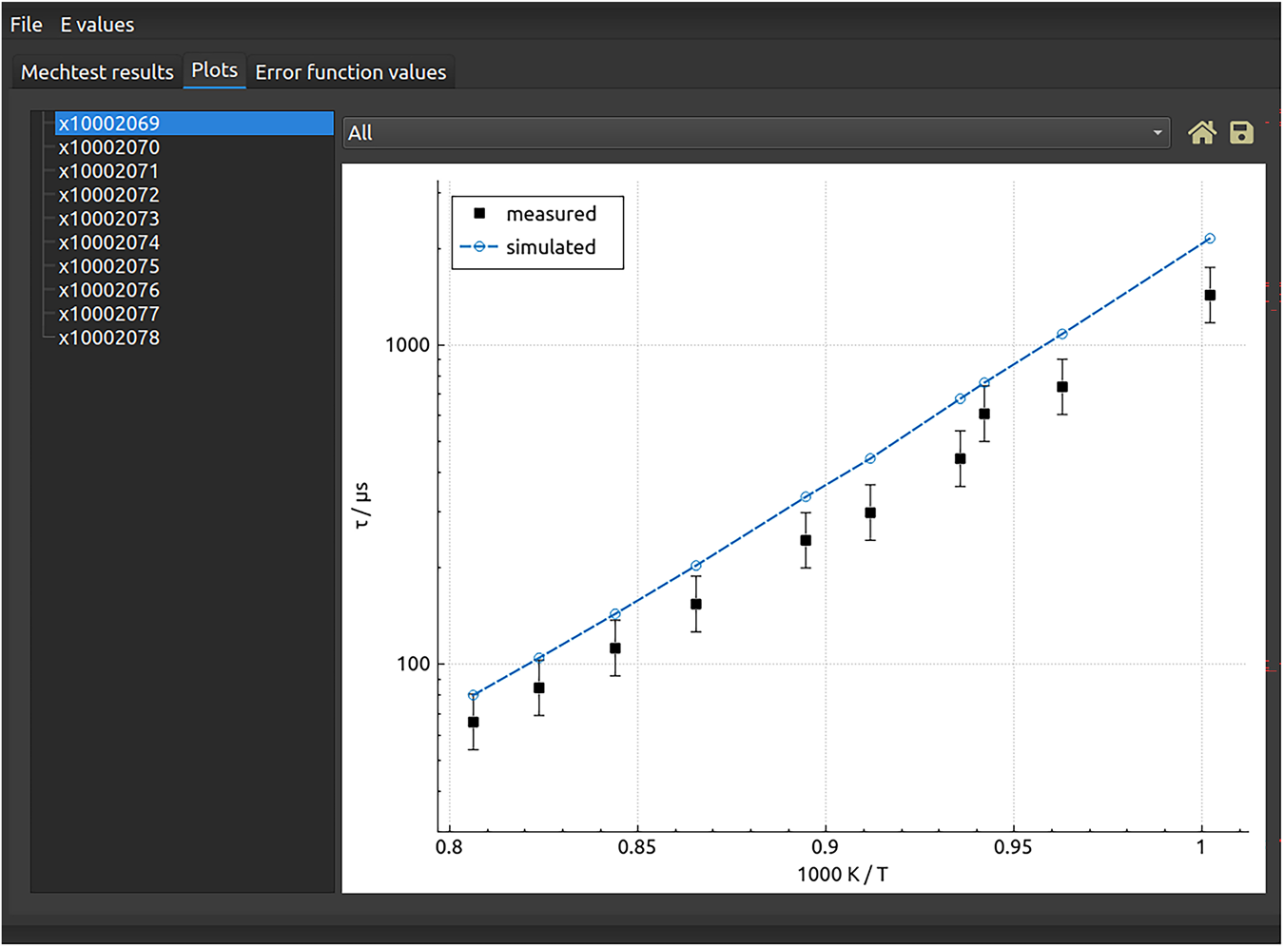
Optima++-generated figure that compares the experimental data (solid squares), the experimental 2*σ* uncertainty (error bars), and the simulation results (dots interconnected by dased lines) The list of data files used are given on the left hand side.

**Fig. 3:**
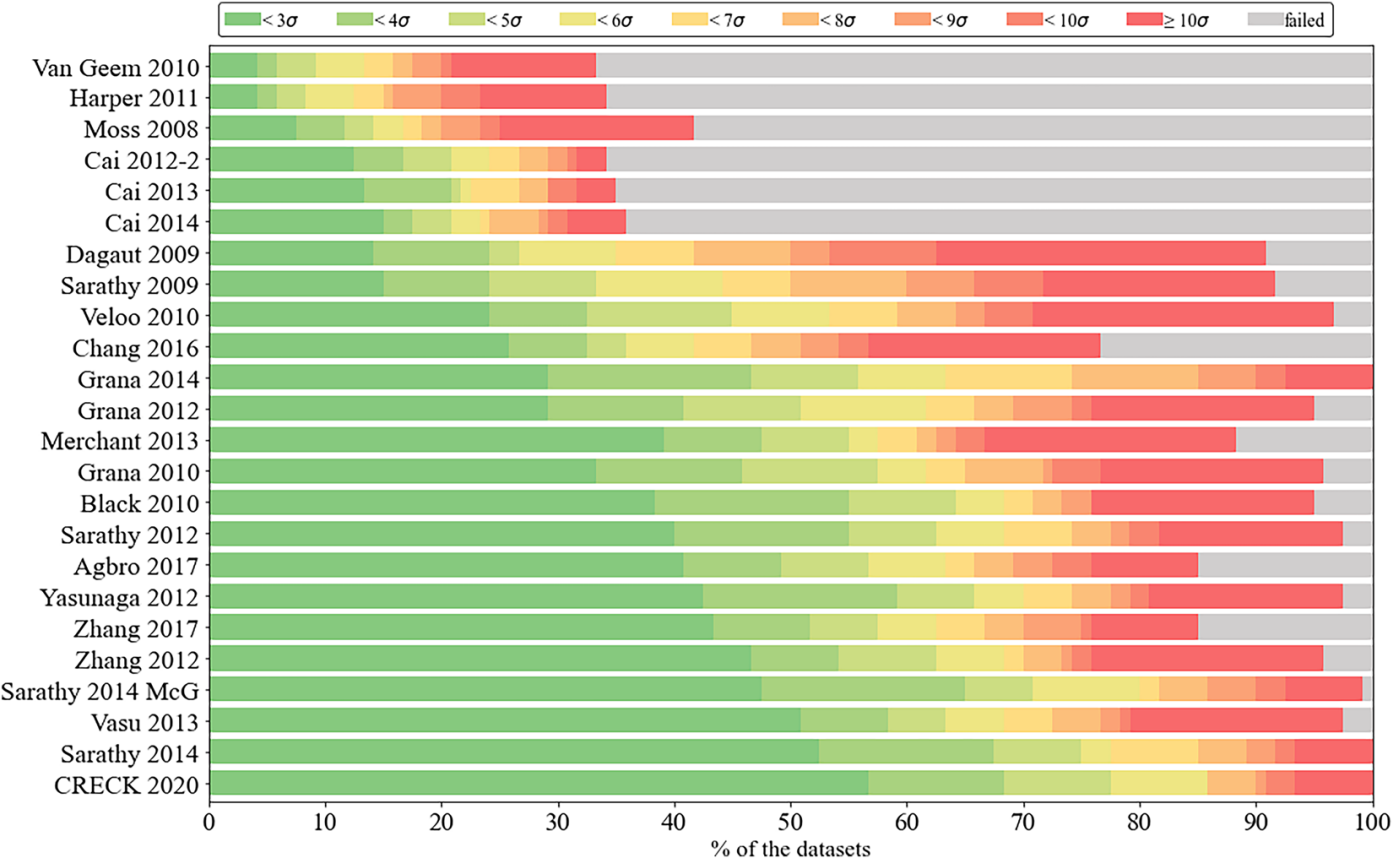
Comparison of the performance of 24 detailed butanol combustion reaction mechanisms on *n*-butanol combustion experimental data (Optima++-generated figure). Various colored bars (olive, green, yellow, and orange) show the percentage of the data points that were reproduced within integer multiples of the experimental uncertainty *σ*. Red bars show the percentage of data that were reproduced higher than 10*σ* uncertainty, while the grey bars show the percentage of failed simulations.

Optima++ is able to test a combustion mechanism against experimental data. It reads the names of the RKD data files to be used and carries out all simulations using a selected mechanism. Using the estimated uncertainty of the experimental data encoded in the RKD format indirect data files, an error function value is calculated that shows the squared difference between the experimental data and the simulation results compared to the variance of the experimental data. Optima++ is also able to create a series of figures which makes a visual comparison of the experimental data and simulation results (see Fig. 2). Figure 3 illustrates the performance of several mechanisms on the same set of experimental data. Optima++ can be used to carry out a local sensitivity analysis; it (a) identifies the most important reaction steps and thermodynamic parameters at each experimental data point, and (b) provides a list of the important reactions and thermodynamic parameters at all data points.

Finally, Optima++ is able to carry out the optimization of detailed reaction mechanisms^41,139,140^. The code fits the Arrhenius *A*, *n*, and *E* parameters of selected reaction steps and certain thermodynamic parameters to selected experimental data. These thermodynamic parameters are NASA polynomial coefficients^141^*a*_1_, *a*_6_, and *a*_7_, which are the temperature-independent parameters of the heat capacity at constant pressure, enthalpy of formation, and molar entropy. Optima++ allows using the FOCTOPUS^140,142^, Nelder–Mead simplex^143^, and BOBYQA^144,145^ algorithms. In our detailed investigations^142^ we have found that FOCTOPUS should be the default method, but the Nelder–Mead simplex and the BOBYQA methods allow fast and accurate optimization in simpler cases, especially when only the Arrhenius *A* parameters are fitted. The optimized mechanism obtained is then to be tested with Optima++ on a wider range of experimental data.

Minimal Spline Fit is a utility code for the calculation of the random noise in an experimental dataset^146^. It determines a minimal-parameter spline that captures the noise-free evolution of a noisy dataset and it estimates the standard deviation of the noise based on the fitted curve. The code fits Akima splines with an increasing number of control points (*m*), and fits polynomials with increasing order (*m* = 0, 1, 2, …). The optimal fitted curve can be deduced from the evolution of the fitting error as a function of the parameters of the fitting curve.

FluxViewer++ is a reaction path visualization tool, which reads the flux information file generated by Optima++. The code draws the species as boxes and the element fluxes as interconnecting arrows. The boxes and arrows can be moved by the mouse to create an aesthetic arrangement. The boxes, the arrows, and the other visualization settings can be saved into a session file for later reuse. The final figure can be saved in commonly used image formats (.png, .jpeg, and .bmp) in a chosen resolution. Development and change of the connections with reaction progress can be inspected as an animation. The frames of the animation can also be saved to create a video or an animated GIF file.

SEM is a mechanism reduction code based on the method of simulation error minimization (SEM)^147,148^. Application of SEM requires significantly more CPU time than that of the widely used DRG or DRGEP methods, but the reduced mechanism found by SEM is much smaller, at the same level of agreement between the full and reduced mechanisms (“simulation error”). Furthermore, the allowed simulation error is an input parameter for SEM.

ReactionKinetics is a Mathematica-based package that investigates reaction mechanisms using a selected set of structural and graph-theoretical approaches, as well as techniques related to the time evolution of the concentration sets obtained from the considered mechanisms. The hydrogen and syngas reaction mechanisms present in the mechanism collection of ReSpecTh have been analyzed^149^ with ReactionKinetics.

### High-resolution spectroscopy

The MARVEL algorithm^34,35,64–66^ is applicable to molecules of arbitrary size and complexity and it was run over the entire set of input spectroscopic data present in ReSpecTh. This involved the following steps. (1) For each molecule, all measured transitions reported in the literature were collected, converted into a predefined format, and compiled into a dataset, serving as the input for the MARVEL code. Although the supplementary information attached to articles nowadays is of considerable help during the addition of dataset entries, the transitions reported in older papers, unfortunately, had to be entered into the dataset manually or with the help of a scanner and a character recognition software. As a result, even a carefully prepared dataset may contain errors and typos which need to be checked and corrected before performing a calculation with the MARVEL code. (2) Using a least-squares method, the best possible set of empirical rovibronic energy values was determined with the help of MARVEL, followed by the validation of the measured lines with these energies. (3) When a discrepancy was found between an observed position and its MARVEL-predicted counterpart, the indicated assignment issue, line-position problem, or underestimated uncertainty was resolved. After resolving a conflict, MARVEL was rerun repeatedly to guarantee that all conflicts were eliminated. At the end of the iterative MARVEL process, self-consistent datasets emerged, that is, ones without internal conflicts. Nevertheless, these may not be correct datasets, as the input data may contain errors that cannot be revealed without additional information. The list of molecules for which spectroscopic datasets were created is given in Table 2.

**Table 2:** Spectroscopic (MARVEL) datasets available within ReSpecTh.

Molecule	Isotopologue	#lines	#levels	Year	Reference
AlH	^27^AlH	682	259	2018	Yurchenko *et al*.^158^
BeH	^9^BeH	2201	1264	2018	Darby-Lewis *et al*.^159^
	^9^BeD	2605	1495	2018	Darby-Lewis *et al*.^159^
	^9^BeT	538	215	2018	Darby-Lewis *et al*.^159^
C_2_	^12^C_2_	31 323	7047	2020	McKemmish *et al*.^68^
CH	^12^CH	6348	1521	2022	Furtenbacher *et al*.^160^
CN	^12^C^14^N	40 516	8083	2020	Syme *et al*.^161^
CP	^12^C^31^P	3264	948	2021	Qin *et al*.^162^
NH	^14^NH	3002	1058	2019	Darby-Lewis *et al*.^70^
NO	^14^N^16^O	11 136	4106	2017	Wong *et al*.^163^
O_2_	^16^O_2_	30 671	15 946	2019	Furtenbacher *et al*.^71^
OH	^16^OH	15 938	1624	2022	Furtenbacher *et al*.^160^
PN	^31^P^14^N	1735	61	2021	Qin *et al*.^164^
TiO	^48^Ti^16^O	56 240	10 761	2019	McKemmish *et al*.^165^
ZrO	^90^Zr^16^O	22 549	8088	2018	McKemmish *et al*.^73^
CaOH	^40^Ca^16^OH	3 240	1 955	2020	Wang *et al*.^166^
H_2_O	${{\rm{H}}}_{2}^{16}{\rm{O}}$	309 290	19 027	2024	Furtenbacher *et al*.^83^
	${{\rm{H}}}_{2}^{17}{\rm{O}}$	27 045	5278	2020	Furtenbacher *et al*.^86^
	${{\rm{H}}}_{2}^{18}{\rm{O}}$	66 166	6865	2020	Furtenbacher *et al*.^95^
	HD^16^O	54 740	8819	2010	Tennyson *et al*.^80^
	HD^17^O	485	162	2010	Tennyson *et al*.^80^
	HD^18^O	8728	1864	2010	Tennyson *et al*.^80^
	${{\rm{D}}}_{2}^{16}{\rm{O}}$	53 534	12 269	2014	Tennyson *et al*.^82^
	${{\rm{D}}}_{2}^{17}{\rm{O}}$	600	338	2014	Tennyson *et al*.^82^
	${{\rm{D}}}_{2}^{18}{\rm{O}}$	12 146	3350	2014	Tennyson *et al*.^82^
H_2_S	H_2_^32^S	44 325	7436	2018	Chubb *et al*.^89^
${{\rm{H}}}_{3}^{+}$	${{\rm{H}}}_{3}^{+}$	1610	652	2013	Furtenbacher *et al*.^76^
	H_2_D^+^	195	86	2013	Furtenbacher *et al*.^76^
	D_2_H^+^	154	72	2013	Furtenbacher *et al*.^76^
HOCl	H^16^O^35^Cl	20 349	5760	2023	Ecseri *et al*.^88^
	H^16^O^37^Cl	10 266	3933	2023	Ecseri *et al*.^88^
SO_2_	^32^S^16^O_2_	40 325	15 130	2018	Tóbiás *et al*.^91^
	^33^S^16^O_2_	15 647	5852	2018	Tóbiás *et al*.^91^
	^34^S^16^O_2_	31 088	10 893	2018	Tóbiás *et al*.^91^
	^36^S^16^O_2_	31	—	2018	Tóbiás *et al*.^91^
NH_3_	^14^NH_3_	29 450	4961	2015	Al Derzi *et al*.^94^
C_2_H_2_	^12^C_2_H_2_	37 813	11 213	2018	Chubb *et al*.^92^
H_2_CO	${{\rm{H}}}_{2}^{12}{{\rm{C}}}^{16}{\rm{O}}$	39 662	5029	2021	Al-Derzi *et al*.^93^
H_2_C_2_O	${{\rm{H}}}_{2}^{12}{{\rm{C}}}_{2}^{16}{\rm{O}}$	3879	353	2011	Fábri *et al*.^96^

As seen in Table 2, we collected and validated more than one million measured transitions. Using these transitions, almost 200 000 rovibronic energy levels were derived.

#### Utility codes

The browser-based custom code MARVELOnline, with a graphical user interface, was used during the collection and validation of the spectroscopic data. It is available at http://kkrk.chem.elte.hu/marvelonline/.

TransitionFileConverter is a tool which converts older versions of a MARVEL input file into the latest one. Over time, there have been several formats for the transition files. Therefore, the need arose for a tool which can be accessed online to convert these older formats to the format required by the current version of MARVEL. A line in the current version of the input transitions file contains the following data: (a) line position, (b) initial uncertainty of the line position, (c) an actual (MARVEL) uncertainty, (d) descriptors, usually rovibrational assignments and symmetry information, characterizing the upper and lower states involved in the transition, and (e) line tag. There is no limit to the number of descriptors used for identifying a state, but each state must have a unique label with the same number of descriptors, separated by a white space. Since from quantum theory MARVEL utilizes only the Ritz principle^62^, it is not required that all descriptors, or even just one, have a clear physical meaning. The line tag provides provenance, it is used for an easy identification of the transitions, and it must be unique for each line in the transition file.

CheckTransitionFile inspects whether the transition file generated by the user meets the requirements of the current MARVEL version. In addition, this tool also investigates the fulfillment of some basic requirements: (a) the initial/actual transition uncertainties need to have nonzero values, (b) the upper and lower states have to be different, and (c) the quantum states in the transition file must have the same number of labels.

Subgraph can display a spectroscopic subnetwork built around a given state. The user can choose the depth of the display, *i.e*., how many neighboring levels should appear on the figure (the maximum value is 10). This tool is particularly useful to understand which transitions and which states affect the energy value of a particular state.

EnergyTable allows the tabulation of the empirical energy values deduced at the end of a MARVEL analysis. It is possible to group these values, and to obtain important statistical information (*e.g*., mean and standard deviations, maximum and minimum values), as well, for the individual groups.

### Thermochemistry

Computation of quantum-chemical energy and enthalpy results, including reaction enthalpies, is based more naturally on the electronic ground states of the atoms and not on the historically preferred elemental states. Since these two possible protocols can be converted into each other straightforwardly, it was proposed, following earlier suggestions^109–113^, that first-principles thermochemistry, like NEAT^114^, standing for a “network of computed reaction enthalpies leading to atom-based thermochemistry”, should employ the ground electronic states of atoms. In atom-based thermochemistry, the enthalpy of formation for a gaseous compound corresponds simply to the total atomization energy (TAE) of that species; it is always positive, and it reflects the bonding strength within the molecule.

The NEAT dataset collected and put in the OSF (Open Science Framework) repository^30^ contains computed reaction enthalpies for 355 reactions. This dataset was used to determine 0 K enthalpies of formation for 188 species.

## Data Records

All ReSpecTh datasets are made publicly available at the following OSF repository:^30 ^https://osf.io/nbmzv/. The datasets of each branch of ReSpecTh are put into separate folders, *i.e*., the top level of the OSF repository contains three folders: *ReactionKinetics*, *Spectroscopy*, and *Thermochemistry*.

### Reaction kinetics

The *ReactionKinetics* folder contains two sub-folders called *indirect* and *direct*. The *direct* folder contains the results of direct experimental or theoretical determination of reaction rate coefficients. These types of experiments determine the rate coefficient of an elementary reaction at a given set of conditions (temperature, pressure, and bath gas).

As to indirect experimental combustion data, collections were created according to fuel and measurement types. A search in the indirect experimental datasets is allowed according to the chemical composition of the initial mixture, experiment type, and the ranges of temperature, pressure, and equivalence ratio. This way, it is straightforward to locate all experimental data within ReSpecTh according to a given set of experimental conditions, which can significantly assist mechanism development. It is also possible to search according to bibliographic data of the experimental publication, such as author names, DOI number, and the year of the publication. All data files found by a search condition can be downloaded in a single step.

At the conditions of the indirect experimental data, the most important reaction steps have been identified for the combustion of the fuels hydrogen, syngas, methanol, ethanol, hydrogen/NO_x_, syngas/NO_x_, methanol/NO_x_, methane, ethylene, and ammonia. The direct experimental and theoretical determinations of these rate coefficients were encoded in RKD format data files. ReSpecTh contains 8451 data points in 443 XML data files in this category. The direct data cover not only high-temperature, but also room-temperature and sub-room-temperature ranges; therefore, these data are also of interest in astronomy or atmospheric chemistry. This collection of experimentally or theoretically determined gas-phase rate coefficients is also used on the so-called k-evaluation website^150^. This is an interactive website that allows the determination of the temperature-dependent uncertainty of the rate coefficients, using both the data stored there and the data added by the user.

Note that both the categories of indirect and direct methods are organized according to the type of information and not the experimental method. For example, the laminar burning velocities are measured by one of the five main experimental approaches and these approaches have several subcategories. Furthermore, the category of direct measurements covers several very different experimental methods. However, the measured quantity is the same and therefore the exact experimental method is indicated only as auxiliary information.

### High-resolution spectroscopy

The folder *Spectroscopy* contains two files: *Spectroscopic_Datasets.zip* and *Spectroscopic_Datasets.pdf*. The spectroscopic datasets are organized into folders, they can be found in the file *Spectroscopic_Datasets.zip*. Each folder contains the experimentally measured transitions, collected for the given molecule, in a text file. The folder also contains, where it is available, the empirical rovibronic energy levels of the molecule. As the transitions measured for each molecule have been labeled in a molecule-specific way, each transition and energy file contains a different number of columns. Thus, before using a spectroscopic dataset, it is recommended to review the reference of the chosen molecule. These references can be found in the file *Spectroscopic_Datasets.pdf*.

The file *Spectroscopic_Datasets.zip* contains information for a total of 23 molecules and 39 isotopologues. Table 2 lists the molecules and the isotopologues treated, together with the number of measured transitions (#lines) collected, the number of empirical rovibrational energy levels (#levels) determined, and the reference to the article where the results were originally published. It is important to note that the OSF repository contains not only the current (latest) version of a given spectroscopic database, but all previous versions are available. For example, for the ${{\rm{H}}}_{2}^{16}{\rm{O}}$ molecule, three datasets are available in the OSF repository: the so-called PART III^81^, W2020^83^, and the latest W2024^86^ dataset.

### Thermochemistry

The folder *Thermochemistry* contains three sub-folders: *AccurateThermochemistry*, *BURCAT*, and *NEAT*. The folder *AccurateThermochemistry* contains accurate thermochemical functions for the ^12^C_2_, D_2_O, ${{\rm{H}}}_{2}^{16}{\rm{O}}$, and ^16^O_2_ molecules, organized into subfolders. Each sub-folder contains a “README” file, which specifies the columns of the thermochemical files, given in text format. The folder *BURCAT* contains Burcat’s Thermodynamic Database, both in text and XML format, and a “README” file, which describes the format of these files. The folder *NEAT* contains two files. The file *NEAT_reactions.txt* contains the full list of reactions in NEAT format. The file header contains the description of the columns. The file *NEAT_enthalpies.txt* lists the computed NEAT enthalpies of the species. This file also has a header showing the column descriptions. For more details, we recommend to consult ref. ^114^.

## Technical Validation

### Reaction kinetics

When direct experimental data are published, the main text of the article, or the supplementary material accompanying the paper, usually includes a table containing the measured rate coefficients, while the text describes the temperature, the pressure, and the bath gas that is related to the rate coefficient. These articles usually contain figures that plot the rate coefficient values as a function of temperature (so-called Arrhenius plots) or pressure (so-called fall-off curves). The contents of the RKD format data files were converted to similar plots and these were compared with the published figures. A similar procedure was followed for the theoretically determined rate coefficients in case the calculated rate coefficients were provided. If only the Arrhenius parameters were published in a theoretical paper, the rate coefficients were calculated at several temperatures and stored in the RKD format data files.

Most of the indirect experimental data, like ignition delay times or laminar burning velocities, were not published in digital form, but provided as dots in the figures. In the case of a newer publication, the person creating the RKD datafile contacted the original authors by e-mail and requested the original values used to create the plots. For earlier publications, these values are typically not available. In this case, the dots of the figures were digitalized. The accuracy of the digitalization was checked in two ways. First, the data in the RKD file were plotted using the Optima++ code and these plots were compared with the figures of the original publication. A complementary route was also followed, making simulations, with the help of the RKD files, with a new mechanism. Most of the data were well reproducible with these simulations.

Both the indirect and direct RKD data files were checked in such a way that the data points were compared to each other and the results of simulations. A small fraction, about 10 %, of the collected data was found to be potentially not reliable. These data were indicated by gray shading in the supplementary tables of our publications.

During the last decade, a part of the reaction kinetics data was collected by undergraduate students. The data files created by them were always systematically checked by PhD students or postdocs. In most cases, the data collected earlier were checked by a data evaluator, comparing them against the original publication.

### High-resolution spectroscopy

A major reason behind the introduction of a network-based approach to high-resolution spectroscopy^34,151^ was the possibility to develop new tools for the validation of the diverse set of experimental high-resolution spectroscopic results present in line-by-line datasets. In fact, the MARVEL algorithm^34,35,64^ does provide several tools itself, based on graph theory^65,66,152^, which help the validation of experimental line positions and the detection of incorrect entries in the list of transitions^84,91,153–155^.

For each molecule reported in Table 2, an extensive validation process was carried out. It was found that the best way to identify incorrect or inaccurate literature entries is offered by the simulation of the measured spectrum, utilizing line positions deduced from the empirical energy levels, augmented with first-principles computed intensities. Given the uncertainties of the computed energy levels and transitions, a decision about the experimental line questioned could usually be made. When in doubt about which line causes the issue, the traditional techniques of experimental spectroscopists, based on combination differences, also proved to be helpful. Each publication, also indicated in Table 2, lists the transitions which had to be deleted during the MARVEL treatment of the data.

Effective Hamiltonian models are designed to act in a reduced space and describe part of the eigenvalue spectrum of a complete Hamiltonian. These models were also used to validate a number of measurements, especially those upon which they are based.

**Fig. 4:**
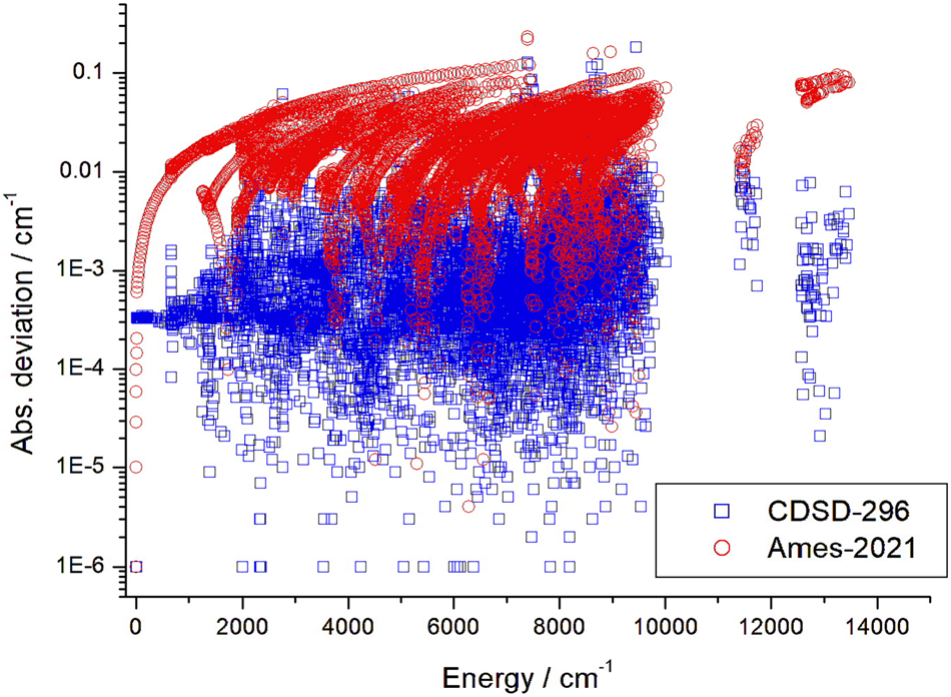
Absolute deviations between the empirical rovibrational energies of the ^16^O^12^C^18^O isotopologue of carbon dioxide obtained through the MARVEL approach^75^ and those of CDSD-296^15^ (blue squares) and Ames-2021^156^ (red circles).

Figure 4 provides an example, for the case of the ^16^O^12^C^18^O isotopologue of carbon dioxide^75^ where rovibrational energies obtained by MARVEL are compared with those of an effective Hamiltonian model, contained in the CDSD-296^15^ database. A comparison is also made there between MARVEL and a set of first-principles computed (Ames-2021^156^) results. As expected and indeed seen in Fig. 4, the effective Hamiltonian model results in much more accurate energy levels than a first-principles computation. Nevertheless, Fig. 4 also shows that at about 7500 and 9000 cm^−1^ there are a number of MARVEL energy levels whose deviation from the EH results are much larger than the average (expected) deviation. In these cases, it is very difficult to decide whether the empirical MARVEL or the computed energies are correct, calling for new measurements, perhaps designed on the basis of network principles^84,157^.

First-principles line lists help to detect problems with experimental datasets, and they were used indeed for the validation of MARVEL input data (for details, see the original publications listed in Table 2).

### Thermochemistry

To check the accuracy of the enthalpies of formation derived within the NEAT approach, they were compared to their counterparts within the Active Thermochemical Tables (ATcT) database^25^. Table 3 shows the comparison between the NEAT data and those of ATcT for a few closed- and open-shell species. Clearly, the two sets of values agree within the uncertainty limits and, as expected, ATcT has significantly tighter bounds.

**Table 3:** Comparison of selected NEAT-based enthalpies of formation with those of the ATcT approach.

Species	NEAT	ATcT
H_2_O	918.034(150)	917.83(3)
C_2_H_2_	1625.826(360)	1626.16(24)
CH	334.675(150)	334.66(23)
NH_3_	1157.341(290)	1157.25(4)
CO	1071.936(260)	1072.13(9)
CH_3_	1209.576(280)	1209.63(13)
CH_2_	752.409(200)	752.70(26)
OH	425.84(150)	425.62(3)
CO_2_	1597.927(370)	1598.27(9)

All values correspond to 0 K and are in kJ mol^−1^. The uncertainties quoted in parentheses are the expanded uncertainties using a coverage factor of 2 (*i.e*., the quoted uncertainty is  ±2*σ* for both datasets).

## Data Availability

No custom code was used to create the datasets available in the OSF repository^30^.
